# Development and Optimization of Osmotically Controlled Asymmetric Membrane Capsules for Delivery of Solid Dispersion of Lycopene

**DOI:** 10.1155/2014/438528

**Published:** 2014-01-29

**Authors:** Nitin Jain, Rashmi Sareen, Neeraj Mahindroo, K. L. Dhar

**Affiliations:** School of Pharmaceutical Sciences, Shoolini University, Bajhol, Solan, Himachal Pradesh 173229, India

## Abstract

The aim of the present investigation is to develop and statistically optimize the osmotically controlled asymmetric membrane capsules of solid dispersion of lycopene. Solid dispersions of lycopene with *β*-cyclodextrin in different ratios were prepared using solvent evaporation method. Solubility studies showed that the solid dispersion with 1 : 5 (lycopene : *β*-cyclodextrin) exhibited optimum solubility (56.25 mg/mL) for osmotic controlled delivery. Asymmetric membrane capsules (AMCs) were prepared on glass mold pins via dip coating method. Membrane characterization by scanning electron microscopy showed inner porous region and outer dense region. Central composite design response surface methodology was applied for the optimization of AMCs. The independent variables were ethyl cellulose (*X*_1_), glycerol (*X*_2_), and NaCl (*X*_3_) which were varied at different levels to analyze the effect on dependent variables (percentage of cumulative drug release (*Y*_1_) and correlation coefficient of drug release (*Y*_2_)). The effect of independent variables on the response was significantly influential. The F_18_ was selected as optimized formulation based on percentage of CDR (cumulative drug release) of 85.63% and correlation coefficient of 0.9994. The optimized formulation was subjected to analyze the effect of osmotic pressure and agitational intensity on percentage of CDR. The drug release was independent of agitational intensity but was dependent on osmotic pressure of dissolution medium.

## 1. Introduction

The most important properties of oral drug delivery system are better patient compliance and ease of administration; due to this, oral delivery systems gain large share of market among various novel drug delivery technologies. For controlled delivery of drugs, the most efficient strategy available is osmotic devices based reliable drug delivery systems [1]. These types of systems work on the principle of osmosis. Osmotic pressure is generated by active material in itself or induced with the help of osmogen. When this system comes in contact with gastrointestinal tract, the fluid enters into the preparation and dissolves the active material in the core [2]. Thus the release of the solution is induced by the pressure created in the preparation at a slow but regular rate. The zero-order drug release kinetics of osmotic drug delivery system is independent on the chemical properties of the drug, physiological factors related to patients, or intake of food [3, 4], and it depends upon osmotic pressure of core components and formulation factors, including solubility of drug within the tablet core, characteristics of semipermeable membrane, and the size of orifice [5]. The asymmetric membrane capsule (AMC) consists of core containing drug material covered by an asymmetric membrane [6, 7]. Higher rate of water influx is one of the main advantages of asymmetric membrane capsule allowing the drug release with lower osmotic pressures or lower solubilities [8]. Lycopene is one of the most common carotenoids found in serum of human being and the major one is in plasma [9, 10]. Among various carotenoids, lycopene is the most potent antioxidant because of the presence of large number of conjugated double bonds [11]. However, high lipophilicity (log *P* = 17.64) of lycopene resulting in its extremely low aqueous solubility acts as barrier to its oral formulation and bioavailability [12].

This study aims to design and optimize osmotically controlled asymmetric membrane capsule of lycopene with enhanced solubility by forming solid dispersion with *β*-cyclodextrin. A three-factor central composite design (CCD) was used to optimize formulations of asymmetric membrane capsules containing solid dispersion of lycopene. The effect of osmotic pressure and agitational intensity on drug release has also been examined in this work.

## 2. Materials and Methods

### 2.1. Materials

Lycopene was obtained as gift sample from Alpha Remedies, Ambala, India. Ethyl cellulose, sodium chloride, and glycerol were procured from S.D. fine chemicals, Mumbai, India. Ethanol, acetone, and glycerol were procured from Nice Chemicals, New Delhi. All the chemicals and solvents used were of analytical grade.

### 2.2. Solubility Studies

The release kinetics of drug through osmotic controlled systems is directly related to the solubility of the drug within the formulation. Therefore, the solubility of the drug in various dissolution media was assessed by taking excess amount of drug in double distilled water (pH 6.8), 0.1 N HCl (pH 1.2), and phosphate buffer (pH 7.4) in orbital solubility shaker (Inco, India) at 37° ± 0.5°C [13]. Excess amounts were taken to ensure saturation and all the solutions were equilibrated for 72 h. After 72 h saturated solutions were filtered, and after suitable dilutions the concentration of drug was determined at 472 nm using a double beam UV spectrophotometer (Shimadzu 1700, Japan).

### 2.3. Solid Dispersion of Lycopene

Solid dispersions of lycopene with *β*-cyclodextrin were prepared by solvent evaporation method. Lycopene and *β*-cyclodextrin were varied in different ratios (1 : 1 to 1 : 5) to achieve the desired solubility. Accurately weighed quantities of the drug and the polymer were separately dissolved in ethanol and the solutions were mixed together after solubilization and then sonicated for 30 min [14]. The solutions were maintained at 40°C for 4 h followed by lyophilization and then the solid dispersions were also assessed for solubility.

### 2.4. Fabrication of Asymmetric Membrane Capsule

The asymmetric membrane capsules (AMCs) were fabricated based on central composite design (Table 1). Dip coating method was used for the preparation of AMCs. The glass mold pins (diameter 5.92 ± 0.07 for body and 6.46 ± 0.05 for the cap) were dipped in polymeric solution of ethyl cellulose (10% w/v and 15% w/v) and glycerol (12% w/v and 15% w/v) dissolved in mixture of ethanol and acetone. The mold pins were allowed to dip in polymeric solution for 20 s. After 20 s the glass mold pins were removed from the polymeric solution and were quenched in aqueous solution of glycerol (10% w/v) for 10 min. After quenching the AMCs were dried for 10 s and then trimmed off for appropriate size from the mold pins. The AMCs were filled manually with lycopene solid dispersion (LSD) and sodium chloride after uniformly mixed in polythene bag. The AMCs were sealed with 10% w/v solution of ethanol in acetone and water.

### 2.5. Characterization of AMCs

The asymmetric membrane capsules were analyzed for physical appearance and geometric dimensions and compared with conventional gelatin capsules.

#### 2.5.1. Scanning Electron Microscopy

AMCs were examined for their asymmetric structure using SEM (QUANTA 250, FEI Makers, Singapore). The asymmetric membrane surfaces (inner and outer) were sputter coated for 5–10 min with gold by using fine coat ion sputter and examined under SEM [15].

#### 2.5.2. *In Vitro* Drug Release

*In vitro* release studies were performed for developed AMCs to evaluate the effect of various formulation parameters on the release rate of the drug. Dissolution was performed in USP-II dissolution apparatus (Electrolab EDT-LX 08, Mumbai) at 37° ± 0.5°C and 100 rpm for 12 h. The dissolution fluid simulated gastric fluid (SGF) (pH 1.2) for first two hours and simulated intestinal fluid (SIF) (pH 7.4) for subsequent hours [16]. Sample (5 mL) was withdrawn periodically and replaced with preheated (37° ± 0.5°C) fresh medium. The samples were analyzed by UV spectrophotometer (Shimadzu 1700) at 472 nm.

### 2.6. Optimization Study

The effect of independent variables such as concentration of ethyl cellulose (*X*_1_), glycerol (*X*_2_), and sodium chloride (*X*_3_) was analyzed by using central composite design. The responses selected for the study were (i) percentage of  *in vitro* cumulative drug release (*Y*_1_), because formulations were designed to release the drug up to 12 h and (ii) correlation coefficient of drug release (*r* ^2^) as formulations were expected to follow zero order release. The experiments were modeled by Design Expert 8.0.5.2 software (Stat-Ease, Inc., Minneapolis, USA). The layout for central composite design is given in Table 1. The selection of optimized formulation was based on maximum values of *Y*_1_ and *Y*_2_. Further the optimized formulation was subjected to analyze the effect of osmotic pressure and agitational intensity on drug release.

### 2.7. Effect of Osmotic Pressure on Drug Release

The release of drug from AMC depends upon osmotic pressure and osmotic agent present inside the formulation. Release studies of the optimized formulation were performed in the media of different osmotic pressure for the assessment of the mechanism of lycopene release. Sodium chloride (osmotically active solute) was used to vary the osmotic pressure of the dissolution medium (SIF) and the pH was adjusted to 7.4 ± 0.5. In these studies formulation contained (i) 100 mg of NaCl inside and 0 mg outside (dissolution medium) and (ii) 100 mg of NaCl inside and 50 mg outside. All the studies were performed in 900 mL of medium using USP-II dissolution apparatus at 100 rpm.

### 2.8. Effect of Agitational Intensity on Drug Release

This study was carried out in a view to analyze the effect of GI motility on the drug release. Release studies of the optimized formulation were carried out in USP-II dissolution apparatus at different rpm (50, 100, and 150), in order to study the effect of agitational intensity on the release of the drug from the AMC. Samples were withdrawn at predetermined intervals and analyzed by UV spectrophotometer at 472 nm.

## 3. Results and Discussion

### 3.1. Solubility Studies

The solubility of lycopene in different medium was 0.1 N HCl (2.23 × 10^−3^ g/cm^3^), double distilled water (3.07 × 10^−3^ g/cm^3^), and phosphate buffer (3.73 × 10^−3^ g/cm^3^). Low solubility of lycopene indicated the need of solubility enhancement, as with low solubility the release of drug from the AMC would be erratic.

### 3.2. Solid Dispersion of Lycopene

The solid dispersions of lycopene with *β*-cyclodextrin (LSDs) in different ratios (1 : 1 to 1 : 5) were successfully prepared by solvent evaporation method. The solubility study of solid dispersion revealed enhanced solubility of drug. The LSD with 1 : 5 (drug : polymer) showed solubility of 56.25 mg/mL (Table 2) and was selected for AMC since for osmotic controlled delivery, the drug should have solubility in the range of 50–300 mg/mL [13].

### 3.3. Characterization of Asymmetric Membrane Capsule

**Figure 1:**
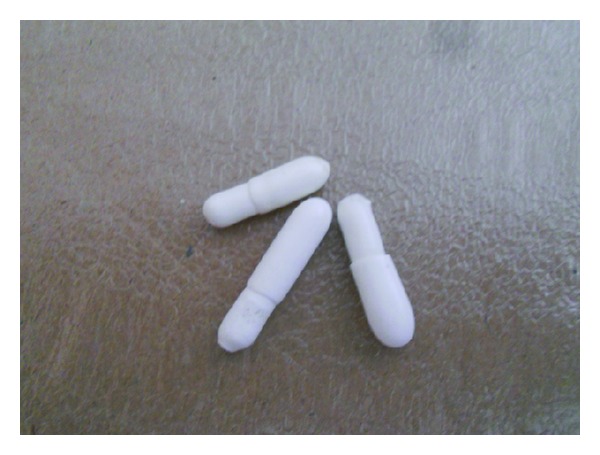
Photograph of asymmetric membrane capsules.

As per the procedure stated above the AMCs (Figure 1) were prepared by dip coating method. AMCs were compared with conventional gelatin capsules (CGCs) for appearance and geometric characterization. AMCs showed higher opacity as compared to CGCs. Comparison of dimensions of AMCs revealed high degree of similarity with CGCs (*P* < 0.005) (Table 3).

### 3.4. Scanning Electron Microscopy

**Figure 2:**
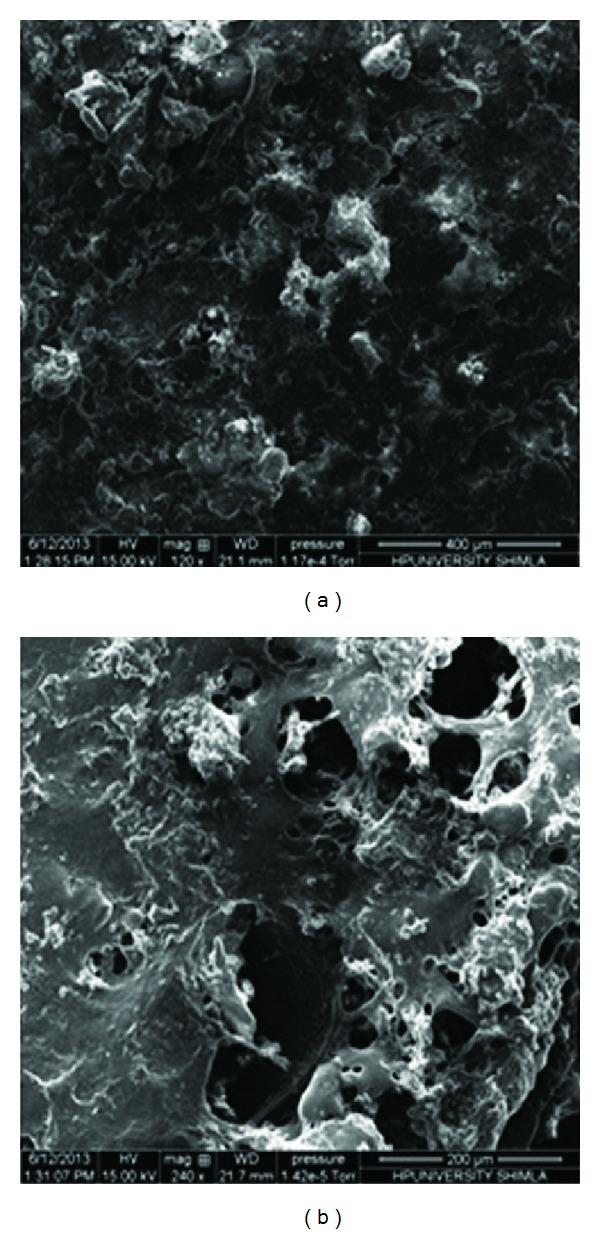
SEM photomicrographs of (a) outer surface and (b) inner surface of AMC.

SEM photomicrographs of developed AMCs showed outer dense region (Figure 2(a)) and inner porous region (Figure 2(b)). SEM photomicrographs revealed the asymmetric nature of capsules which is desirable for osmosis. The asymmetric nature of membrane may be due to incorporation of plasticizer (glycerol), which acted as pore former.

### 3.5. Optimization Study

The effect of independent variables over the dependent variables was assessed by using central composite design.

#### 3.5.1. Influence of Independent Variables on Cumulative Percentage of Drug Release (*Y*_1_)

**Figure 3:**
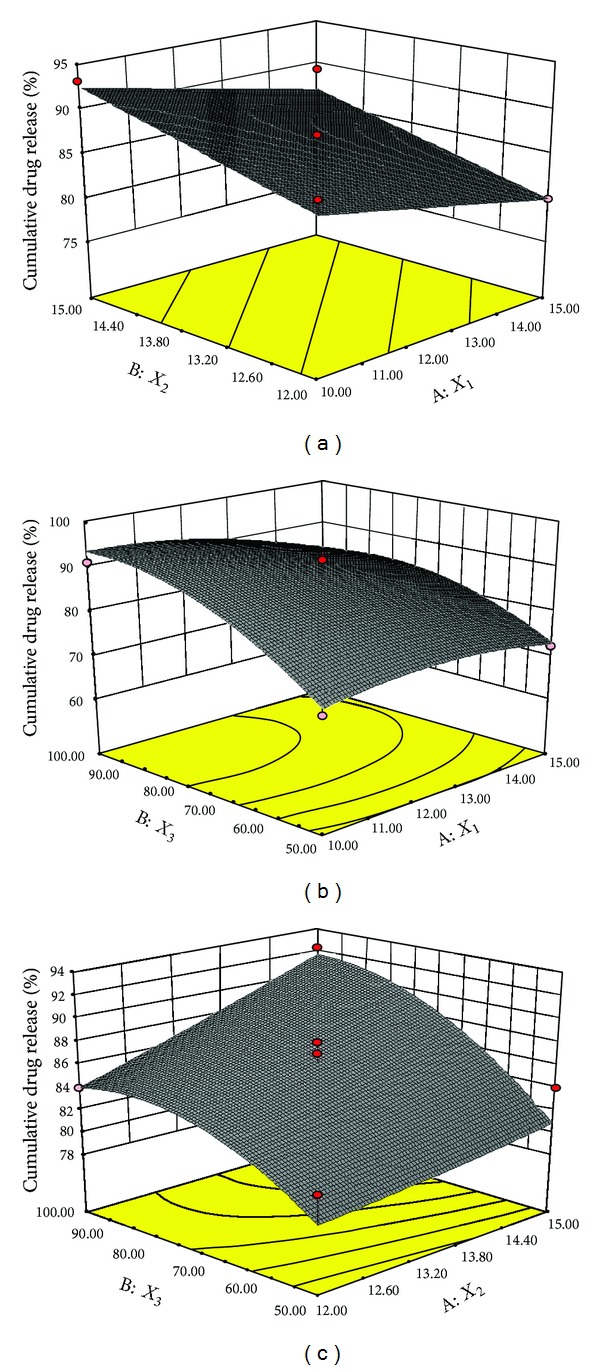
3D-response surface plots for percentage of cumulative drug release as functions of two factors.

The results of *in vitro* release suggested decrease in the release of drug with increase in the level of ethyl cellulose (EC) (*X*_1_). This may be due to the fact that with higher level of EC, the drug has to travel more path length. Due to this reason higher concentration of EC impedes the drug release (Figure 3(a)).  Figure 3(c) demonstrated that release of drug from the system increased with the increase in level of glycerol. The reason may be that higher level of glycerol (*X*_2_) will result in more porous structure of inner layer of AMCs and higher porosity of inner surface resulted in higher drug release. NaCl (*X*_3_) also affected the release rate in the manner that on increasing the level of NaCl the release rate was found to increase due to higher osmotic pumping effect caused by high concentration of osmogen (Figure 3(b)).

#### 3.5.2. Influence of Independent Variables on Correlation Coefficient of Drug Release (*Y*_2_)

**Figure 4:**
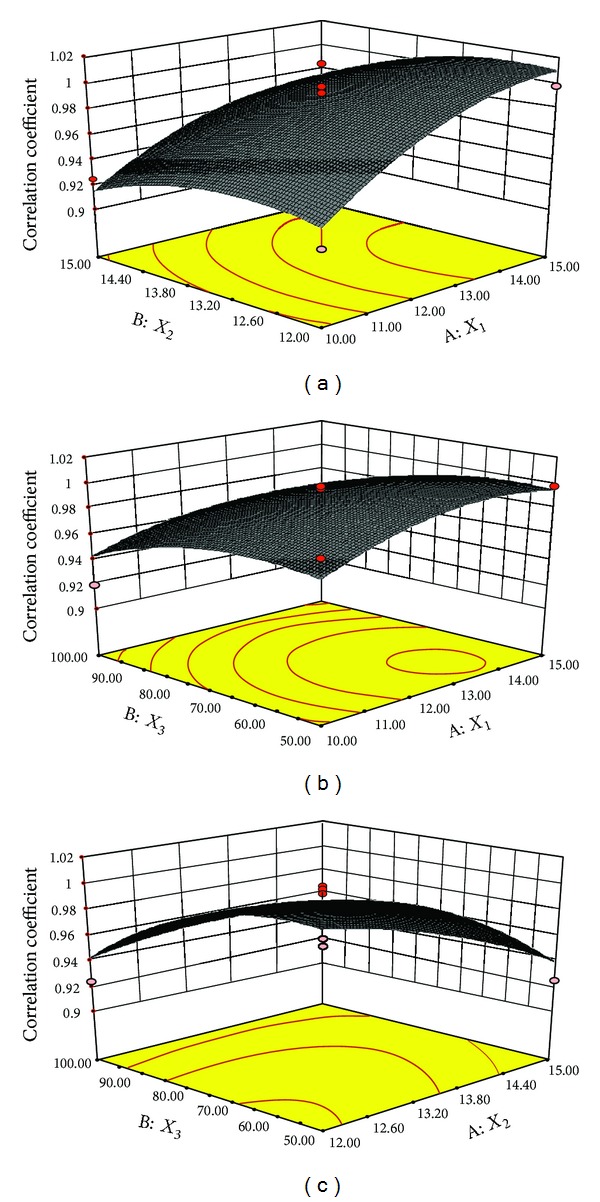
3D-response surface plots for correlation coefficient as functions of two factors.

The correlation coefficient was found to increase with increase in the level of *X*_1_ as evident form Figure 4(a). Results suggested that increase in level of *X*_2_ caused decrease in *Y*_2_ (Figure 4(c)). The reason behind this may be that the increase in level of *X*_2_ resulted in more porous inner structure of AMC which resulted in irregular drug release.  Figure 4(b) showed increase in *Y*_2_ with increase in the level of *X*_3_ which was followed by decrease in *Y*_2_. This may be due to the reason that increase in level of NaCl provided adequate osmotic pressure making hydration of core of capsule and resulted in gentle release profile.

#### 3.5.3. Selection of Optimized Formulation

The optimized formulation was selected on the basis of maximum value of percentage of cumulative drug release (*Y*_1_) and correlation coefficient of drug release (*Y*_2_). F_18_ was selected as optimized formulation having 85.63% of drug release and 0.9994 of correlation coefficient of drug. Further the F_18_ was subjected to analyze the effect of osmotic pressure and agitational intensity on *in vitro* drug release.

### 3.6. Effect of Osmotic Pressure on *In Vitro* Drug Release

**Figure 5:**
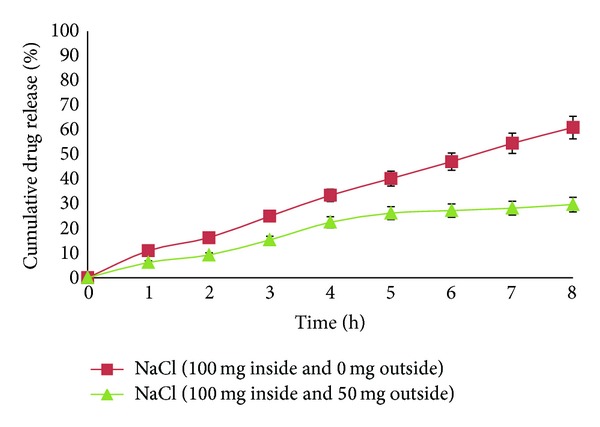
Comparative *in vitro* dissolution profiles showing effect of osmotic pressure.

Release studies of lycopene from AMC were performed to analyze mechanism of drug release. Results indicated that the release was highly dependent on osmotic pressure of release medium. Lycopene release from AMC with 100 mg of NaCl inside and 0 mg outside showed controlled release due to creation of molar environment by osmogen (NaCl). The AMC with 100 mg of NaCl inside and 50 mg outside exhibited slow release with plateau reaching in the sixth hour (Figure 5). This was may be due to the decreased osmotic gradient. All the results suggested osmotic pumping as a primary mechanism of drug release from AMC.

### 3.7. Effect of Agitational Intensity on *In Vitro* Drug Release

**Figure 6:**
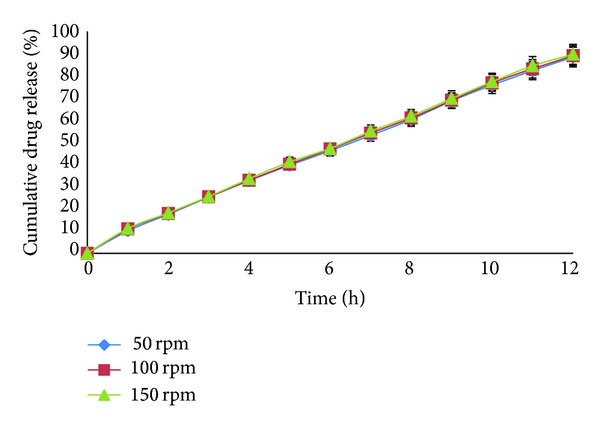
Dissolution profile of F_18_ showing effects of agitational intensity.

Figure 6 showed the release of lycopene from AMC at three different rpm (50, 100, and 150). It can be seen that the lycopene release from AMC was independent of agitational intensity of dissolution media as there was nonsignificant difference in release of drug at different rpm (*P* < 0.01, *f*_2_ = 98.17).

## 4. Conclusion

Asymmetric membrane capsule for the solid dispersion of lycopene with *β*-cyclodextrin was successfully developed using dip coating method and optimized using central composite design. The developed system was able to display complete drug delivery and zero-order release rate. Optimization studies proved that central composite design is efficient for understanding influence of formulation factors on the drug release behaviors. Results concluded that the release of optimized formulation (F_18_) was dependent upon the osmotic pressure of dissolution medium while there was no effect of agitational intensity on the release of drug.

## Figures and Tables

**Table 1:** Experimental design for three factors and experimental values of responses.

Formulation code	*X*_1_ (ethyl cellulose) % w/v	*X*_2_ (glycerol) % w/v	*X*_3_ (NaCl) mg	*Y*_1_ (% cumulative drug Release)	*Y*_2_ (correlation coefficient)
F_1_	12.50	13.50	75.00	89.26	0.9923
F_2_	12.50	13.50	117.04	94.39	0.9825
F_3_	10.00	15.00	100.00	93.74	0.9245
F_4_	10.00	12.00	50.00	79.65	0.9820
F_5_	12.50	13.50	32.96	71.20	0.9714
F_6_	8.30	13.50	75.00	92.45	0.9526
F_7_	12.50	13.50	75.00	89.96	0.9918
F_8_	12.50	13.50	75.00	90.41	0.9921
F_9_	10.00	15.00	50.00	83.63	0.9245
F_10_	12.50	13.50	75.00	90.41	0.9951
F_11_	12.50	13.50	75.00	92.57	0.9829
F_12_	15.00	12.00	50.00	69.47	0.9975
F_13_	16.70	13.50	75.00	74.11	0.9986
F_14_	15.00	15.00	50.00	81.84	0.9842
F_15_	15.00	15.00	100.00	85.90	0.9198
F_16_	10.00	12.00	100.00	89.65	0.9185
F_17_	12.50	13.50	75.00	91.76	0.9914
F_18_	12.50	10.98	75.00	85.63	0.9994
F_19_	15.00	12.00	100.00	75.26	0.9982
F_20_	12.50	16.02	75.00	90.32	0.9725

**Table 2:** Solubility study of solid dispersions of lycopene.

Lycopene : *β*-cyclodextrin	Solubility (mg/mL)
1 : 1	9.29 ± 1.06
1 : 2	22.68 ± 0.72
1 : 3	30.19 ± 0.84
1 : 4	39.78 ± 2.49
1 : 5	56.25 ± 1.26

**Table 3:** Geometric and physical characterization of AMC* as compared to CGC*.

Type of capsule	Appearance	Dimensions, mm				
		Cap		Body		Sealed
		Length	Diameter	Length	Diameter	
CGC	Transparent	9.25 ± 0.18	5.91 ± 0.09	15.98 ± 0.15	5.21 ± 0.09	20.15 ± 0.08
AMC	Opaque	9.37 ± 0.22	6.02 ± 0.11	16.07 ± 0.27	5.37 ± 0.13	20.27 ± 0.16

*AMC indicates asymmetric membrane capsule and CGC indicates conventional gelatin capsule.
